# Supplementary material for: Renormalization group theory for percolation in time-varying networks
Source: Sci Rep. 2018 May 22;8:8011. doi: 10.1038/s41598-018-25363-2 (PMC5964206; doi:10.1038/s41598-018-25363-2)
Supplement: Supplementary file 1 — Supplementary information [file 41598_2018_25363_MOESM1_ESM.pdf]

Supplementary Information

Renormalization group theory for percolation in time-varying networks

Jens Karschau[1], Marco Zimmerling[1], and Benjamin M Friedrich[1,*]

[1]cfaed, TU Dresden, 01069 Dresden, Germany
[*]benjamin.m.friedrich@tu-dresden.de

Numerical methods

For simulations, we considered a time-continuous version of our dynamic network model, where each link can switch between an active and an inactive state with respective rates $r_A = r_{A\to I}$ and $r_I = r_{I\to A}$. This time-continuous formulation of the model, suitable for fast computations using a Gillespie algorithm, is directly related to the time-discrete process studied analytically above: choosing a finite sampling time Δt defines transition probabilities $q_A = p(I, \Delta t | A, 0)$ and $q_I = p(A, \Delta t | I, 0)$ with

$$q_I = \Phi[1 - \exp(-(r_I + r_A)\Delta t)], \tag{S1}$$
$$q_A = (1 - \Phi)[1 - \exp(-(r_I + r_A)\Delta t)]. \tag{S2}$$

Effective rates r'_A and r'_I for switching of network state are determined as the inverse mean of the distribution of waiting times, which define network transition rates q'_I and q'_A analogous to Eqs. (S2).

Variance of reliability estimator

We outline the derivation of Eq. (5) for the variance of the estimate Φ_{est} for the reliability Φ of a link. We will perform the calculation in the time-continuous domain for ease of calculation, and then derive an approximation for the time-discrete case. According to Eqs. (S1-S2), we introduce transition rates $r_A = -\Phi \ln \beta / \Delta t$, $r_I = -(1-\Phi) \ln \beta / \Delta t$, and consider a time-continuous Markov chain that switches between two states I and A with rates r_A (for $I \to A$) and r_I (for $A \to I$), respectively. We introduce a stochastic process $S(t)$ that takes the value 1 if the Markov chain is in state A at time t and 0 else. The expectation value of $S(t)$ equals the reliability by detailed balance

$$\langle S(t) \rangle = \frac{r_A}{r_A + r_I} = \Phi. \tag{S3}$$

The auto-correlation function of $S(t)$ can be computed using path-integral techniques[1]

$$\langle S(0)S(t) \rangle = \frac{\Phi}{r_A + r_I} \left(r_A + r_I \exp[-(r_A + r_I)|t|] \right). \tag{S4}$$

The observation of $S(t)$ in a time interval $[0, T]$ allows to estimate the reliability Φ as

$$\Phi_{\text{est}} = \frac{1}{T} \int_0^T dt\, S(t). \tag{S5}$$

We are interested in the variance of this estimate

$$\begin{aligned} \langle \Phi_{\text{est}}^2 \rangle - \langle \Phi_{\text{est}} \rangle^2 &= \int_0^T dt_1 \int_0^T dt_2 \, \langle S(t_1) S(t_2) \rangle - \Phi^2 \\ &= \frac{2}{T} \frac{r_A r_I}{(r_A + r_I)^3} \left(1 - \frac{1 - \exp[-(r_A + r_I)T]}{T(r_A + r_I)} \right). \end{aligned} \tag{S6}$$

In the limit $(r_A + r_I)\Delta t \ll 1$, corresponding to $1 - \beta \ll 1$, this exact result represents a good approximation also for the time-discrete case. Fig. S1 compares Eq. (S6) to simulation results. The leading-order term in Eq. (S6) scales with the inverse of the number n of subsequent time steps observed and is reported in Eq. (5) in the main text.

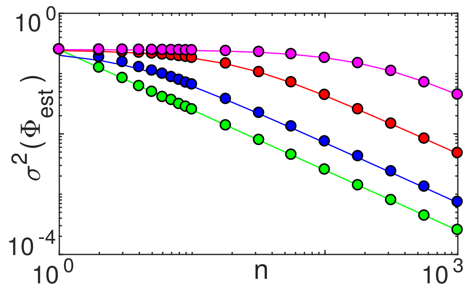

Figure S1. Estimation error for link reliability in the presence of temporal correlations. Shown is the variance of the estimated reliability Φ_{est} as a function of the number $n = T/\mathrm{d}t$ of subsequent time-steps observed for different values of the Bernoulliness β (circles: green $\beta = 0$, blue $\beta = 0.50$, red $\beta = 0.90$, magenta $\beta = 0.99$). Additionally, we show the analytical result Eq. (4) of the main text for the case $\beta = 0$ (green line), as well as the approximative result Eq. (S6) for the case $\beta > 0$ (solid lines, colors according to colors of circles).

Distribution of switching rates

The RG approach is also valid if the switching rates are drawn from a distribution with given mean and variance, as shown in Fig. S2. In this case, also the variance of the distribution becomes renormalized. For example, we find for the variance of the network reliability $\sigma^2(\Phi') = (\partial\Phi'/\partial\Phi)^2\ \sigma^2(\Phi) = 4\Phi^2(1-\Phi)^2[2+5\Phi(1-\Phi)]^2\ \sigma^2(\Phi)$. Correspondingly, the variance increases upon renormalization for $\Phi \approx \Phi_c$, yet decreases for either $\Phi \approx 0$ or $\Phi \approx 1$.

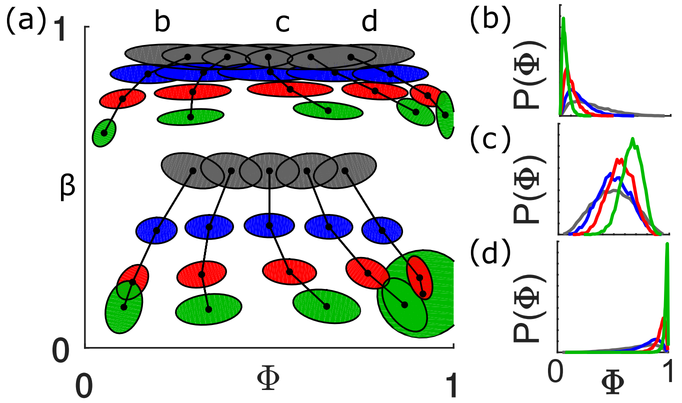

Figure S2. Renormalization of a triangular lattice motif with link rates q_A and q_I drawn from a log-normal distribution. (a) Flow in (Φ, β)-parameter space under the action of the renormalization group. The covariance ellipses enclose a probability of 2/3 each. (b-d) Distribution of motif reliability for selected cases in (a), using the same color-code for motifs of increasing size.

Case of non-zero processing delay

In our minimal model, we assumed that transmission and processing delays of messages are much shorter than the time scale at which links switch their state. Here, we relax this assumption and consider a non-zero processing delay $\Delta t_{\mathrm{delay}}$: each message is assumed to spend some time $\Delta t_{\mathrm{delay}}$ at a node, before the message is instantaneously relayed along active links to the neighbor nodes. Additionally, we explicitly state the sending interval t_{send} at which the source node injects messages into the network. Importantly, two cases can be considered: In case (a), the sending interval scales similarly as the processing time. We chose $t_{\mathrm{send}} = t_{\mathrm{delay}}$, for simplicity. In case (b), we consider a hypothetical scenario, where the sending interval remains constant, $t_{\mathrm{send}} = \Delta t$, independent of the processing time t_{delay}.

We find that in case (b), the processing delay has practically no effect on the network Bernoulliness β', see Fig. S3(b). In contrast, in case (a), we find a strong dependence of β' on t_{delay}, see Fig. S3(a). This effect can be attributed to large extent to the increased sending interval. For a single link, theory predicts $\beta(m) = \beta(1)^m$, where $m = t_{\mathrm{send}}/\Delta t$; to good approximation, this relation also holds for network motifs (dashed line).

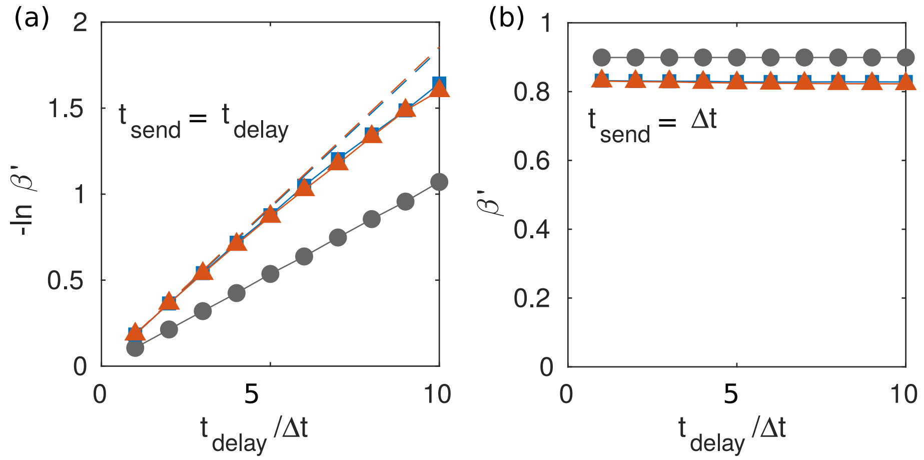

Figure S3. Influence of processing delay. (a) We consider a non-zero processing delay $t_{\text{delay}} = m\Delta t$, and sending interval $t_{\text{send}} = t_{\text{delay}}$. Shown are simulation results for the network Bernoulliness β' for a single link (gray), as well as for the 4-link motif (blue squares: $\Phi = 0.2$; orange triangles: $\Phi = 0.8$). The decrease in Bernoulliness is approximately described by a scaling relation $\beta(m) = \beta(1)^m$ with $m = t_{\text{send}}/\Delta t$ valid for a single link (dashed lines, colors correspond to those of simulation results). (b) Same as in panel (a), except for the sending interval is now kept constant, $t_{\text{send}} = \Delta t$. In this case, t_{delay} has practically no effect on the network Bernoulliness β'.

The cubic lattice

For the renormalization of the cubic lattice, we chose a coarse-graining motif consisting of 4 unit cells with a total of $n = 33$ links as is shown in Fig. S4(a). This choice ensures that the sub-lattice is again a cubic lattice (in this case with three-times the original lattice constant), see Fig. S4(b). For the computation of the renormalized reliability Φ', it suffices to count the number $N_A(n_A)$ of active states of the motif that have exactly n_A active links for $0 \le n_A \le n$,

$$\Phi' = \sum_{n_A=0}^{n} N_A(n_A)\Phi^{n_A}(1-\Phi)^{n-n_A}\,. \tag{S7}$$

Fig. S4(d) displays the number $N_A(n_A)$ of active motif states, and the number $N_I(n_A)$ of inactive motif states. For the computation of the renormalized Bernoulliness β', we first write Eq. (9) in the main text explicitly as

$$q_I' = \sum_{0\le n_A\le n,\,0\le n_A'\le n,\,0\le n_{A\to I}\le n} N(n_A,n_A',n_{A\to I})\, q_I^{n_{A\to I}}(1-q_I)^{n_{A\to A}}\, q_A^{n_{I\to A}}(1-q_A)^{n_{I\to I}}\,\Phi^{n_A}(1-\Phi)^{n_I} \Big/ \sum_{0\le n_A\le n} N_A(n_A)\,\Phi^{n_A}(1-\Phi)^{n_I}\,. \tag{S8}$$

Here, $N(n_A,n_A',n_{A\to I})$ denotes the number of pairs (s,s') of network states, such that $s\in S_A$ is an active motif state with exactly n_A active links (and $n_I = n - n_A$ inactive links), $s'\in S_I$ is an inactive motif state with exactly n_A' active links (and $n_I' = n - n_A'$ inactive links), and the number of links that are active in s but inactive in s' equals $n_{A\to I}$. Additionally, we use short-hand notation $n_{A\to A} = n_A - n_{A\to I}$, $n_{I\to A} = n_A' - n_A + n_{A\to I}$, and $n_{I\to I} = n_I - n_{I\to A}$, to indicate the number of links that switch or retain their state in a transition $s\to s'$ as indicated by the subscript, see also Fig. S4(c). Substituting $q_I = (1-\Phi)(1-\beta)$ and $q_A = \Phi(1-\beta)$ in Eq. (S8) as well as in the analogous equation for q_A' provides β' as a function of Φ and β.

Exact computation of $N(n_A,n_A',n_{A\to I})$ is computationally not feasible. Instead, we estimated the polynomial coefficients of the rational function $\beta' = \beta'(\Phi,\beta)$ by considering $5.1\cdot 10^{11}$ random pairs (s_A,s_I) consisting of active and inactive network states, s_A and s_I, respectively (out of a total of more than $1.8\cdot 10^{19}$ of such pairs), which required a computation time of approximately 48 hours on twelve 3-GHz cores. A comparison of semi-analytical and simulation results is shown in Fig. S4(e).

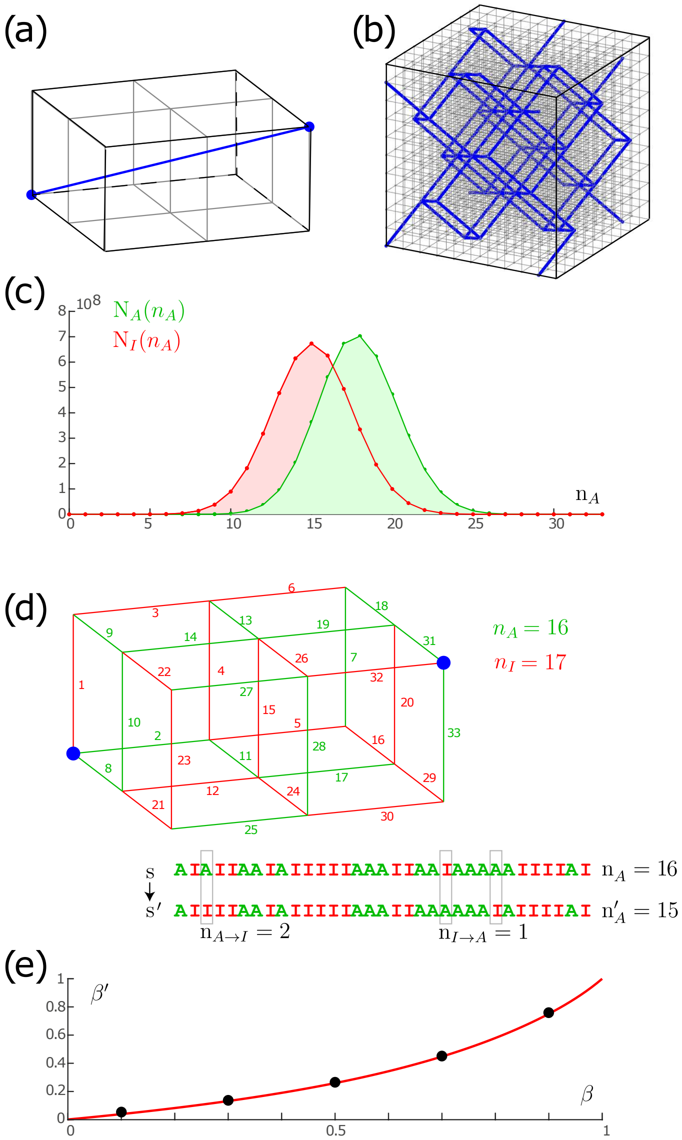

Figure S4. Renormalization of cubic lattice. (a) Coarse-graining motif, consisting of 4 cubic unit cells. (b) The coarse-grained lattice (blue) shown inside the original lattice (gray). (c) Number $N_A(n_A)$ and $N_I(n_A)$ of active and inactive motif states, respectively, as function of the number n_A of active links. (d) Example of motif state. (e) Renormalized Bernoulliness β' as function of link Bernoulliness β for $\Phi = 0.5$, where the critical reliability $\Phi_c \approx 0.50$ according to our RG calculation (solid line: semi-analytical results, points: numerical simulation).

End-to-end reliability and Bernoulliness

We state the analytical expression for the end-to-end reliability Φ' and end-to-end Bernoulliness β' for the coarse-graining motif of the triangular network shown in Fig. 2(b)

$$\Phi' = \Phi^2[(\Phi-2)\Phi(2\Phi-1)+2] \tag{S9}$$

$$\beta' = \sum_{i=1}^{5} \alpha_i \beta^i \tag{S10}$$

where

$$\begin{aligned}
\alpha_1\lambda &= 20\Phi^6 - 60\Phi^5 + 44\Phi^4 + 12\Phi^3 - 12\Phi^2 - 4\Phi,\\
\alpha_2\lambda &= -40\Phi^6 + 120\Phi^5 - 114\Phi^4 + 28\Phi^3 + 6\Phi^2 - 2,\\
\alpha_3\lambda &= 40\Phi^6 - 120\Phi^5 + 128\Phi^4 - 56\Phi^3 + 10\Phi^2 - 2\Phi,\\
\alpha_4\lambda &= -20\Phi^6 + 60\Phi^5 - 65\Phi^4 + 30\Phi^3 - 5\Phi^2,\\
\alpha_5\lambda &= 4\Phi^6 - 12\Phi^5 + 12\Phi^4 - 4\Phi^3,\\
\lambda &= ((\Phi-2)\Phi(2\Phi-1)+2)(\Phi(\Phi(2\Phi-1)-2)-1).
\end{aligned}$$

Similarly, we have for the honeycomb lattice

$$\Phi' = \Phi^3\left(2-\Phi^3\right), \tag{S11}$$

$$\beta' = \sum_{i=1}^{6} \alpha_i \beta^i \tag{S12}$$

with

$$\begin{aligned}
\alpha_1\lambda &= 6\Phi^7 + 6\Phi^6 + 6\Phi^5 - 6\Phi^4 - 6\Phi^3 - 6\Phi^2,\\
\alpha_2\lambda &= -15\Phi^7 + 12\Phi^4 - 6\Phi,\\
\alpha_3\lambda &= 20\Phi^7 - 20\Phi^6 - 4\Phi^4 + 4\Phi^3 + 2\Phi - 2,\\
\alpha_4\lambda &= -15\Phi^7 + 30\Phi^6 - 15\Phi^5,\\
\alpha_5\lambda &= 6\Phi^7 - 18\Phi^6 + 18\Phi^5 - 6\Phi^4,\\
\alpha_6\lambda &= -\Phi^7 + 4\Phi^6 - 6\Phi^5 + 4\Phi^4 - \Phi^3,\\
\lambda &= \left(\Phi^2+\Phi+1\right)^2\left(\Phi^3-2\right),
\end{aligned}$$

and for the square lattice

$$\Phi' = \Phi^2\left(2-\Phi^2\right), \tag{S13}$$

$$\beta' = \sum_{i=1}^{4} \alpha_i \beta^i \tag{S14}$$

with

$$\begin{aligned}
\alpha_1\lambda &= 4\Phi^4 + 4\Phi^3 - 4\Phi^2 - 4\Phi,\\
\alpha_2\lambda &= -6\Phi^4 + 4\Phi^2 - 2,\\
\alpha_3\lambda &= 4\Phi^4 - 4\Phi^3,\\
\alpha_4\lambda &= -\Phi^4 + 2\Phi^3 - \Phi^2,\\
\lambda &= (\Phi+1)^2\left(\Phi^2-2\right).
\end{aligned}$$

Analytical expressions for the triangular, and cubic lattice are not presented due to their length.

References

1. Chaichian, M. & Demichev, A. *Path Integrals in Physics: Volume I Stochastic Processes and Quantum Mechanics* (Insitute of Physics Publishing, 2001).
